# Characterization of a Chitin-Binding Protein from *Bacillus thuringiensis* HD-1

**DOI:** 10.1371/journal.pone.0066603

**Published:** 2013-06-18

**Authors:** Naresh Arora, Bindiya Sachdev, Rani Gupta, Y. Vimala, Raj K. Bhatnagar

**Affiliations:** 1 International Center for Genetic Engineering and Biotechnology, Aruna Asaf Ali Marg, New Delhi, India; 2 Department of Microbiology, University of Delhi, South Campus, New Delhi, India; 3 Department of Botany, Ch. Charan Singh University, Meerut, India; University of Hyderabad, India

## Abstract

Strains of *Bacillus thuringiensis* produce insecticidal proteins. These strains have been isolated from diverse ecological niches, such as soil, phylloplane, insect cadavers and grain dust. To effectively propagate, these strains produce a range of molecules that facilitate its multiplication in a competing environment. In this report, we have examined synthesis of a chitin-binding protein and evaluated its effect on fungi encountered in environment and its interaction with insecticidal proteins synthesized by *B. thuringiensis*. The gene encoding chitin-binding protein has been cloned and expressed. The purified protein has been demonstrated to interact with Cry insecticidal protein, Cry1Ac by Circular Dichrosim spectroscopy (CD) and *in vitro* pull down assays. The chitin-binding protein potentiates insecticidal activity of bacillar insecticidal protein, Cry1Ac. Further, chitin-binding protein was fungistatic against several soil fungi. The chitin binding protein is expressed in spore mother cell and deposited along with insecticidal protein, Cry1Ac. It interacts with Cry1Ac to potentiate its insecticidal activity and facilitate propagation of *Bacillus* strain in environment by inhibiting growth of certain fungi.

## Introduction

Certain strains of gram-positive soil bacterium, *B. thuringiensis* (Bt) produce insecticidal proteins during sporulation. Strains of Bt have been isolated from diverse ecological niches. Spores of these strains contain crystalline inclusions that consist of insecticidal proteins amongst other proteins [1]. The high abundance and prevalence of Bt strains is attributed to its ability to survive a range of environmental conditions and rapid multiplication of spores. The wide occurrence is probably also a consequence of its ability to assimilate nutrients from diverse complex macromolecules including chitin [2]. Chitin is degraded by chitinases (E.C.3.2.1.14) and the resultant product, N-acetyl glucosamine is used as a nutrient. Since strains of Bt successfully grow and multiply on insect cadavers, they secrete a highly active chitinase to degrade chitin [2].

Apart from producing insecticidal proteins, strains of Bt are also known to produce extracellular macromolecules such as lipases, chitinases, proteases, α-exotoxins that facilitate its virulence and successful propagation. Of these macromolecules, S-layer protein has been shown to be insecticidal against beetle [3]. In addition, chitinase produced by Bt was shown to potentiate the insecticidal activity of vegetative insecticidal protein (*vip*) [2]. Together with chitinases, several bacteria, viruses and parasites are known to secrete chitin-binding protein (CBP) upon their growth on chitin as sole source of carbon. At present, the biological function and usefulness of CBPs is not clear but they are believed to facilitate microbial attachment to chitin for its subsequent degradation [4]. CBPs have been shown to act synergistically with chitinases in degradation of natural chitin variants in *Serratia proteamaculans* 568 [5]. CBPs from different microbes display divergence in their preference to chitin binding. Such diversity has been summed up adequately by Purushotham *et al.* [5]. Bacterial CBPs and their binding preferences to chitin have been extensively studied in *Enterococcus faecalis* [6], *Lactococcus lactis* [7], *Thermobifida fusca* [8], *Serratia marcescens* [9].

We have investigated the occurrence of CBP in Bt (Strain-HD1) and examined its binding properties with (a) exochitinase [2] (b) chitin extracted from peritrophic matrix of larvae of insect pest, *Helicoverpa armigera* and (c) insecticidal crystal protein, Cry1Ac.

## Materials and Methods

### Ethics Statement

The animal studies described below were approved by the ICGEB Institutional Animal Ethics Committee (IAEC Reference No. IR-07). ICGEB is licensed to conduct animal studies for research purposes under the registration number 18/1999/CPCSEA (dated 10/1/99). All efforts were made to minimize animal suffering.

### Bacterial Strains, Fungal Strains and Culture Conditions

Bt-HD1 and *E. coli* expressed Cry1Ac toxin were obtained by Bacillus Genetic Stock Center (Ohio State University, Columbus, USA). The Cry1Ac toxin was cloned and expressed in pKK223-3 vector (Amersham) and it does not contain 6X His-tag. Transformants of *E. coli* BL21(DE3) containing pET32-*bt-cbp21* were grown at 37°C in LB broth containing 100 µg ampicillin ml^-1^ [10]. Fungal strains, *Curvularia oryzae, Aspergillus oryzae, Aspergillus parasiticus* and *Verticillium dahliae,* used for the determination of biological activity of Bt-CBP21 were grown on Potato-dextrose medium [11]. These fungal strains were obtained from collection of Prof. Rani Gupta, Department of Microbiology, University of Delhi, South Campus, New Delhi, India).

### Extraction and Purification of Bt-CBP21 from *B. thuringiensis*

Bt-HD1, was grown aerobically in G-medium [12] supplemented with colloidal chitin (0.15%, w/v) as the sole source of carbon. The culture was grown at 30°C for 72 h at 200 rpm and kept stationary for 30 min. The sedimented chitin was washed thrice with phosphate-buffered saline (1X PBS) containing NaCl (0.9%, w/v) and centrifuged. The pellet was finally resuspended in 5 M guanidine-HCl and incubated at 37°C for 30 min with gentle stirring. The suspension containing the chitin-bound proteins was centrifuged at 18,415×g for 20 min at 25°C. The supernatant was dialyzed against 50 mM Tris-HCl, pH 7.5 at 4°C with three buffer changes and concentrated using Centricon Plus-10 concentrator (Millipore). The concentrated sample was passed through a Sephacryl S-100 HR column (Amersham). Elution was performed at a flowrate of 0.5 ml/min in 50 mM Tris-HCl, pH 7.5 containing 150 mM NaCl. Fractions of 1 ml were collected and 0.2 ml of each fraction was precipitated by 10% trichloroacetic acid and analyzed on 10% SDS-PAGE.

### N-terminal Amino Acid Sequencing

The 21 kDa CBP (Bt-CBP21) purified from Bt*-*HD1 and its tryptic digest were fractionated by SDS-PAGE and transferred onto an Immobilon-P PVDF membrane (Millipore) in a semi-dry transfer chamber as directed by the manufacturer. The blotted protein was excised from the membrane and N-terminal sequencing was performed at the Biotechnology Research Laboratory, Medical University of South Carolina, Charleston, USA.

### Cloning of *B. thuringiensis* cbp21

Chromosomal DNA of Bt-HD1 was isolated as described earlier [13]. Based on the N-terminal sequence of purified Bt-CBP21 and sequence of its tryptic peptide, forward and reverse degenerate primers, CBP-F and CBP-R were designed and PCR was performed using chromosomal DNA as template. A 300 bp amplified fragment was cloned into pGEM-Te vector (Promega) and sequenced. Identical sequences were obtained from ten independent clones. Based on the sequence of the cloned insert, gene-specific forward and reverse primers, SpeCBD-F (Biotinylated), SpeCBD-R (Biotinylated) and SpeCBP-F2, SpeCBP-R2 were designed and a PCR-based directional genome walking was performed [14]. The PCR product was cloned into pGEM-Te vector and sequenced to obtain the full-length *bt-cbp21*. The cDNA sequence of *bt-cbp21* has been submitted in NCBI [GenBank: HQ324111].

### Computer-based Sequence Analysis

The deduced amino acid sequence of Bt-CBP21 was used to locate the signal peptide using the SignalP program (http://www.cbs.dtu.dk/services/SignalP/). The domain search was performed using Pfam database at the Sanger Institute, UK (http://www.sanger.ac.uk/Software/Pfam/). The Phylogenetic tree of Bt-CBP21 with other CBPs and proteases was constructed using the ClustalW program (MacVector 7.0). Homology search was done at the NCBI using the BLAST program. DNA sequence analysis was performed with MacVector 7.0.

### Expression of Recombinant Bt-CBP21 in E. coli

The recombinant plasmid carrying the *bt-cbp21* was digested with *EcoR1* and sub-cloned into pET32b (Novagen). Recombinant plasmid was transformed into BL21(DE3) host cells and induced with 1 mM IPTG. The cells were harvested by centrifugation and lysed by sonication on ice (3×30 sec, 100W output) with intermittent cooling. The sonicated suspension was centrifuged at 18,415×g for 15 min at 4°C and the supernatant was collected for further processing.

### Purification of Recombinant Bt-CBP21 and Production of Polyclonal Antibodies

Affinity purification of 6X His recombinant Bt-CBP21 from the soluble fraction was carried out with Ni-NTA agarose (Qiagen) under native conditions with slight modifications. The IPTG induced culture was harvested, washed with ice-cold buffer (50 mM sodium phosphate; pH 8.0) and resuspended in 10 mL buffer A (50 mM sodium phosphate, pH 8.0, 300 mM NaCl) containing 10 mM imidazole. The culture was further incubated on ice for 30 min. The cells were lysed by sonication and the supernatant was mixed with Ni-NTA resin, pre-equilibrated with buffer A at 4°C for 1 h. The resin was then washed with 10 bed volumes of buffer A containing 20 mM imidazole and bound proteins were eluted in buffer A containing 150 mM imidazole. All fractions were analyzed on 10% SDS-PAGE. The purified protein was used for affinity assays and for raising polyclonal antibodies against Bt-CBP21.

Antiserum was raised by immunization of Balb/c mice with 1 mg of purified Bt-CBP21 administered subcutaneously in Freund’s complete adjuvant (Sigma). The mice were boosted twice with purified Bt-CBP21 administered in Freund’s incomplete adjuvant (Sigma) and the reactivity was assessed by Western blotting. The antisera raised against Bt-CBP21 did not cross react with Cry1Ac toxin.

### Preparation of Colloidal Chitin

The protoxin was purified to near homogeneity by procedure described earlier [15]. Colloidal Chitin was prepared from Crab-shell chitin (Sigma) as described earlier [16].

### Chitin-binding Assay for Bt-CBP21 and Cry1Ac

The chitin-binding assay was performed using colloidal chitin as the affinity matrix. Bt-CBP21 (50 µg) was mixed with colloidal chitin (0.15%, w/v) and incubated at 37°C for 30 min and allowed to sediment. The supernatant containing the unbound protein was collected and the pellet was washed several times with 1X PBS containing NaCl (0.9%, w/v) and centrifuged. The chitin-bound proteins were released by boiling the affinity matrix in SDS-sample buffer and resolved on 10% SDS-PAGE.

For assessing binding of Cry1Ac with colloidal chitin, the colloidal chitin was mixed with purified protoxin and eluted in buffers with following pH: 50 mM sodium-acetate, pH 5.5, 50 mM Tris-HCl, pH 7.5 and 50 mM sodium-carbonate, pH 10.5. Chitinase from *B. thuringiensis* (Bt-Chitinase) and *H. armigera* (Ha-Chitinase) were purified as described earlier [2], [17]. The chitinase was assayed using fluorometric substrate, 4-methylumbelliferyl-β-D-N, N’-diacetylchitobioside (Sigma) [17].

### Localization and Immunodetection of Bt-CBP21 in Cultures of Bt-HD1

Renograffin gradient was used to separate spores and parasporal crystals of Bt*-*HD1 from vegetative cells as described earlier [18]. The parasporal crystal-protein complex was resolved on 12% SDS-PAGE and electroblotted onto an ECL nitrocellulose membrane (Amersham). The blot was probed with mouse anti-Bt-CBP21 antibody (1∶30,000), washed with 1X PBS containing 0.1% Tween-20 (PBS-T) and developed with NBT-BCIP substrate (Roche).

### Circular Dichroism of Interaction of Bt-CBP21 and Cry1Ac

Purified proteins, Bt-CBP21 and Cry1Ac (150 µg) were dissolved in 10 mM sodium-phosphate buffer, pH 7.0 and CD spectra analysis were performed at 24°C using a quartz cuvette with a path length of 0.2 cm and recorded in a spectrophotometer (J-800, Jasco). Samples were scanned five times at 50 nm/min with bandwidth of 1 nm and response time of 0.5 sec. The average value was determined for each spectrum and the spectrum of the ‘buffer alone’ served as control. All measurements were conducted twice.

### Protease Assay

The proteolytic activity of purified Bt-CBP21 (both native and recombinant protein) was assayed using azocasein or synthetic substrates, BAPNA (*N*_α_-Benzoyl-L-arginine 4-nitroanilide hydrochloride; Sigma) [19] and SAAPF (Succinyl-Ala-Ala-Pro-Phe-p-nitroaniline; Sigma) [20]. Varying amounts of the purified protein were incubated with suitable substrate and the reaction was monitored as described earlier [21]. The validity of assay reaction was confirmed by including protease purified from *Anopheles culicifacies* as described earlier [21] and chitinase from *H. armigera* [17].

### *In vitro* Pull Down Assay of Cry1Ac and Bt-CBP21

Purified protoxin, Cry1Ac and recombinant Bt-CBP21 were purified as described in the previous section. In a total reaction volume of 100 µl, purified Cry1Ac (40 µg) and Bt-CBP21 (30 µg) were mixed in 1X binding buffer [25 mM Tris-Cl (pH 8.0), 75 mM NaCl, 2.5 mM EDTA, 5 mM MgCl_2_, 1% NP-40, 2.5 mM DTT] followed by incubation at 25°C for 1 h. The protein mixture was mixed with pre-equilibrated Ni-NTA resin (Qiagen) and binding was done with slow agitation on end-over-end mixer for 30 min at 4°C. Unbound proteins were removed upon centrifugation at 835×g for 5 min at 4°C. The resin was washed extensively with 1X binding buffer. Bound proteins were eluted using 1X binding buffer containing 300 mM imidazole. All fractions were analyzed on 10% SDS-PAGE.

### Antifungal Assay

Agar diffusion assays were performed to determine the antifungal activity of Bt-CBP21 and to characterize its spectrum of biological activity. *In vitro* testing of Bt-CBP21 for antifungal activity on *C. oryzae, A. oryzae, A. parasiticus and V. dahliae* was performed with approximately 100 spores of fungi which were seeded into test plates (6-well culture plates) containing 2 ml of Potato-dextrose medium. Upto 20 µg of purified Bt-CBP21 was spotted at the center of the plate and incubated at 25°C and inhibition was monitored for 2 days. Each assay was replicated thrice in triplicates.

### Effect of Bt-CBP21 on *Helicoverpa armigera* Neonates

The culture of *H. armigera* was maintained in the laboratory under controlled conditions of temperature 25°C, 70% relative humidity and a photoperiod of 12 h light:12 h dark. The larvae were reared on a semi-synthetic artificial diet. Bioassays were conducted on neonates by placing them on artificial diet containing purified protoxin, Cry1Ac (≤LC_50_ = 15 ng/cm) and Bt-CBP21 (20 µg/ml). All assays were replicated thrice and 20 larvae were used per treatment. Mortality was recorded 48 h post-treatment.

### T-Test

P-value of synergistic interactions between Bt-CBP21 and Cry1Ac were calculated in terms of T-Test based on the mortality in bioassays conducted on *H. armigera*.

Bioassays were set up in three replicas of 20 insects each on laboratory-reared insects fed on artificial diet. Cry1Ac was used at values ≤ LC50 value and Bt-CBP21 was used at concentrations of 20 µg per replica.

## Results and Discussion

**Figure 1:**
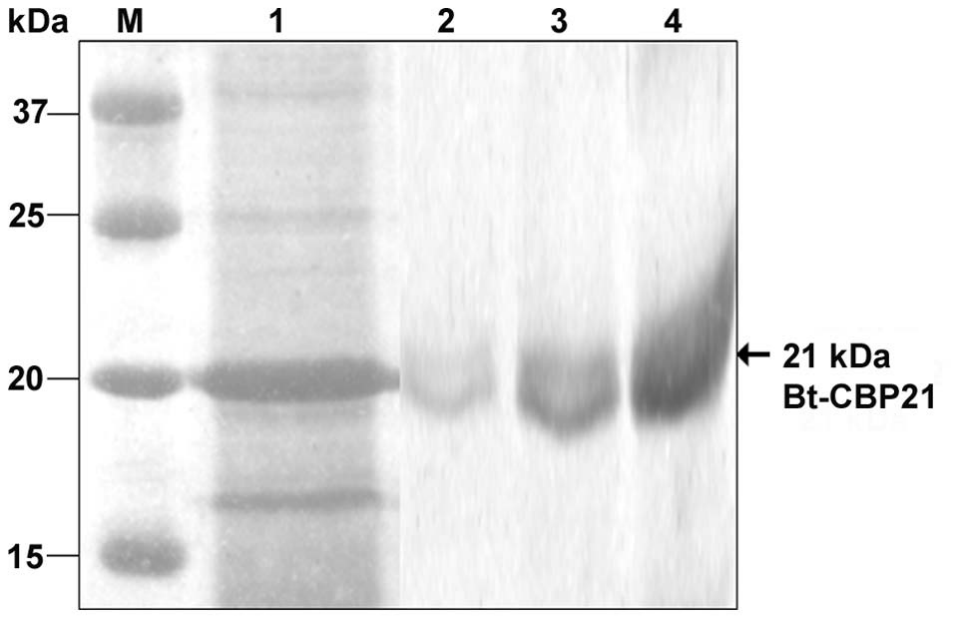
Isolation of native Bt-CBP21 and purification by gel-filtration chromatography. Bt*-*HD1 was grown in G-medium containing colloidal chitin for 72 h at 30°C. The culture was held stationary for 30 min. The settled chitin was washed with 1X PBS containing 0.9% NaCl and centrifuged. The settled chitin was resuspended in 5 M Guanidine-HCl and centrifuged. The supernatant was dialyzed, concentrated by centricon column and passed through gel-filtration column (Sephacryl S-100 4R). Bound proteins were eluted in 50 mM Tris-HCl, pH 7.5 containing 150 mM NaCl. All fractions were resolved on 10% SDS-PAGE. Lane M: Protein standard marker (in kilodaltons), Lane 1: Chitin-bound proteins (crude), Lane 2–4: Fractions containing purified Bt-CBP21.

The synthesis of Bt-CBP21 in Bt*-*HD1 was induced in G-medium containing chitin as the sole source of carbon. Bt-CBP21 was not produced in cultures grown in medium containing glucose as carbon source [2]. Time-course for the synthesis of Bt-CBP21 was followed upto 96 h. The Bt-CBP21 synthesis started after 24 h growth and reached maximum at 72 h of growth. The insoluble chitin matrix was sedimented and the bound proteins were eluted with 5 M Guanidine hydrochloride (Figure 1). Dialysis of the solubilized protein and its resolution through gel-filtration matrix yielded a near homogenous preparation of Bt-CBP21. The N-terminal and internal peptide sequence of Bt-CBP21 was obtained by Edman degradation. The N-terminal sequence (FFSDKEVSNNTFAAGTLD) and sequence of an internal peptide (EPVYETTLADLQK) were used to design primers for the amplification of CBP21 encoding gene, *bt-cbp21*. The PCR amplified product was cloned, sequenced and expressed in *E. coli*.

*In silico* sequence analysis of *bt-cbp21* revealed a polypeptide of 189 residues with a predicted molecular mass of 21 kDa and a pI of 4.51. The SIGNALP program predicted a hydrophobic signal peptide of 19 residues, with cleavage between Ala-19 and Phe-20. The domain search program at Pfam database, classified Bt-CBP21 as a member of chitin binding domain class of proteins (Chitin_bind_3, Pfam entry no. PF03067). The multiple alignment of Bt-CBP21 was performed with 11 other proteins from the chitin-binding domain and a novel surface metalloproteinase, Camelysin. While, Bt-CBP21 exhibited maximum similarity (95%) with camelysin of *Bacillus cereus,* nearly 50% similarity with previously identified chitin binding protein, CHB1 from *Bacillus anthracis, Bacillus halodurans, B. cereus and Oceanobacillus iheyensis* (Figure S1). The Bt-CBP21 and camelysin are nearly identical except for a stretch of 9 amino acid residues at the N-terminal that were absent in Bt-CBP21. The alignment of Bt-CBP21 with other CBPs revealed positional conservation of residues, tryptophan, asparagine, glycine and histidine. The Bt-CBP21 carries a tyrosine residue (Y) corresponding to its location to the W-127 residue in the *B. cereus* camelysin (EMBL accession no. Q816G5). It is interesting that the five additional tryptophan residues in Bt-CBP21 correspond to their relative positions to those present in other spore coat associated proteins of different bacilli. The conserved tryptophan residues have been speculated to be involved in the specific recognition and binding to α-chitin [4]. The absence of cysteines precludes disulfide bond formation in Bt-CBP21 suggesting the flexibility of the protein as compared to other CBPs. The carbohydrate interaction of Bt-CBP21 may be a consequence of several aromatic residues specifically, tyrosine and tryptophan that are speculated to be involved in stacking against the pyranosyl rings of N-acetylglucosamine residues present in chitin [22], [23].

**Figure 2:**
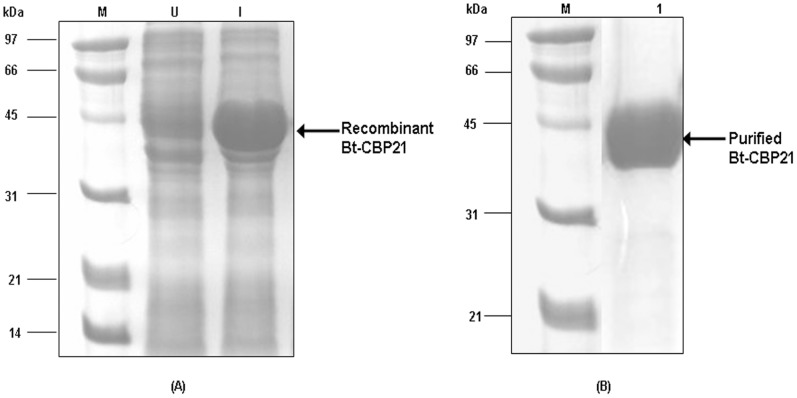
Expression of recombinant Bt-CBP21 in *E.* *coli* and purification by affinity chromatography. (**A**) The recombinant plasmid containing pET32b+*bt-cbp21* was transformed into BL21(DE3) host cells and induced with 1 mM IPTG at 37°C for 2 h. The cells were harvested upon centrifugation and lysed by sonication (3×30 sec; 100W output). The cell suspension was centrifuged and supernatant was collected. Both Induced (Lane I) and uninduced (Lane U) supernatant was resolved on 10% SDS-PAGE. The gel was stained with coomassie brilliant blue. (**B**) The recombinant plasmid containing Bt-CBP21 was IPTG induced and centrifuged as mentioned in Materials and methods. The cells were lysed by sonication in buffer A (50 mM sodium phosphate, pH 8.0; 300 mM NaCl) containing 10 mM Imidazole. The supernatant was loaded onto Ni-NTA resin and washed with buffer A containing 20 mM Imidazole. Bound proteins were eluted in buffer A containing 150 mM imidazole (Lane 1) and resolved on 10% SDS-PAGE. The gel was stained with coomassie brilliant blue.

**Figure 3:**
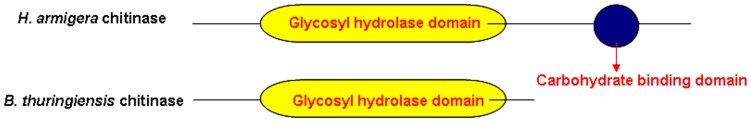
Structural features of chitinase of *H.* *armigera* [17] and *B. thuringiensis* [2].

**Figure 4:**
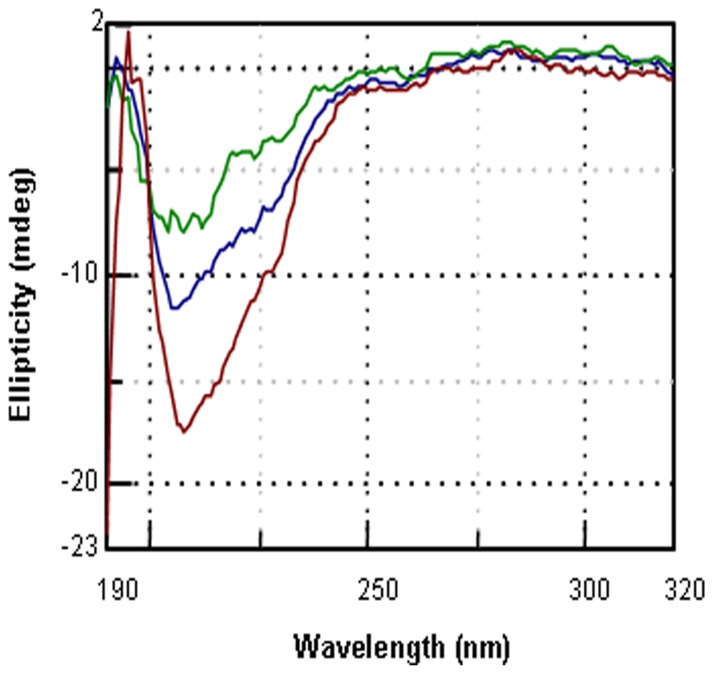
CD spectra of Bt-CBP21 and Cry1Ac toxin. Purified Bt-CBP21 and Cry1Ac (150 µg) were dissolved in 10 mM sodium-phosphate buffer, pH 7.0. Only Bt-CBP21 (Green curve), only Cry1Ac (Blue curve) and mixture of both the proteins (1∶1) (Brown curve) was analysed by CD spectra at 24°C. Samples were scanned five times at 50 nm/min with bandwidth of 1 nm and response time of 0.5 sec in a spectrophotometer (J-800, Jasco). An overlay of each spectrum is shown and spectrum of ‘buffer alone’ served as control. All measurements were conducted twice.

**Figure 5:**
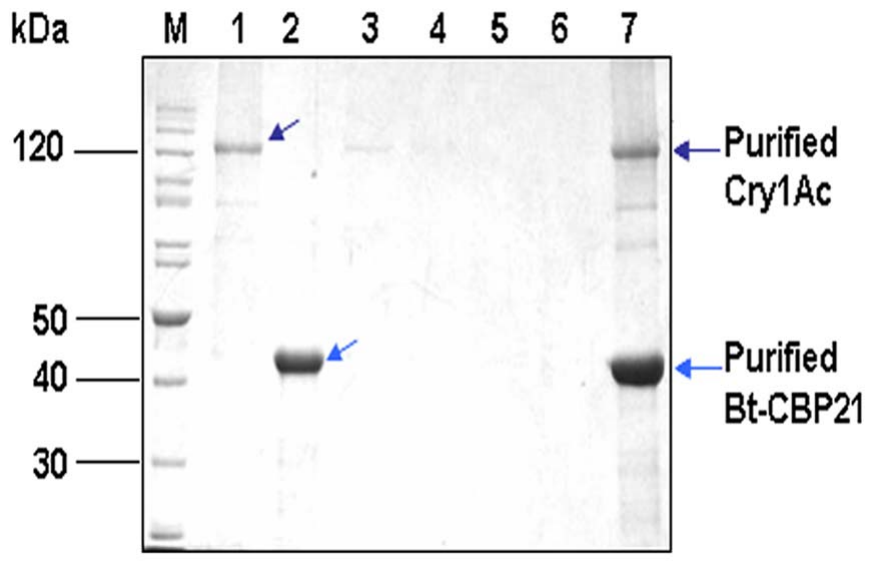
*In vitro* pull down assay of Cry1Ac and Bt-CBP21. Reaction mixture (100 µl) containing purified Cry1Ac protoxin (40 µg) and purified Bt-CBP21 (30 µg) were mixed with 1X binding buffer and incubated for 1 h at 25°C, as described in Materials and methods. Unbound proteins were removed upon centrifugation. The protein mixture was incubated with Ni-NTA matrix for 30 min at 4°C. The resin was washed with 1X binding buffer and bound proteins were eluted with 1X binding buffer containing 300 mM imidazole. All fractions were resolved on 10% SDS-PAGE. Lane M: Protein molecular marker (in kDa), Lane 1: Purified Cry1Ac, Lane 2: Purified Bt-CBP21, Lane 3: Flowthrough, Lanes 4–6: Wash fractions, Lane 7: elution fraction (300 mM Imidazole).

**Figure 6:**
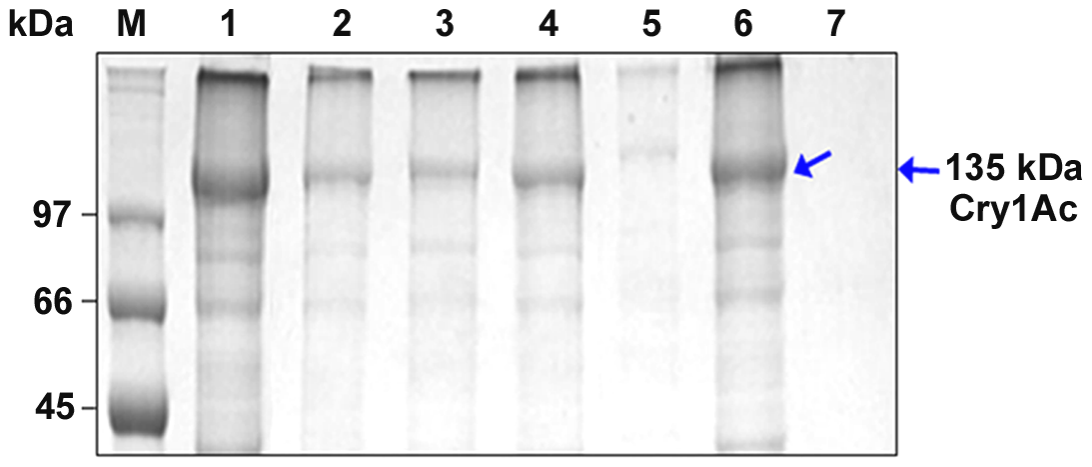
Binding of Cry1Ac protoxin to colloidal chitin. Cry1Ac protoxin was analyzed for its affinity towards colloidal chitin. Protoxin purified on Renograffin gradient was solubilized with 50 mM sodium-carbonate buffer, pH 10.5, passed through PD10 desalting column and bound proteins were eluted with 50 mM sodium-acetate pH 5.5; 50 mM Tris-HCl, pH 7.5 and 50 mM sodium-carbonate, pH 10.5. Proteins were incubated with colloidal chitin isolated from crab shells and pupae caracasses of *H. armigera*. Chitin bound proteins were recovered by boiling the matrix in SDS-sample buffer. All fractions were resolved on 10% SDS-PAGE. Lane M: Protein standard marker (in kilodaltons), Lane 1: Loaded sample, Lanes 2 and 3: Flowthrough and elution fractions at pH 10.5, Lanes 4 and 5: Flowthrough and elution fractions at pH 7.5, Lanes 6 and 7: Flowthrough and elution fractions at pH 5.5.

Since Bt-CBP21 displayed extensive homology with metalloprotease, camelysin of *B. cereus*, we examined the purified Bt-CBP21, native (Figure 1) and recombinant protein (Figure 2A and 2B) for proteolytic and chitinolytic activities. Assays with azocasein or fluorometric substrates revealed absence of proteolytic and chitinolytic activities of Bt-CBP21. Based on the observed structural features of Bt-CBP21, we examined its binding with bacterial and insect chitinases. Both bacterial and insect chitinases were purified to homogeneity and the effect of Bt-CBP21 on their catalytic efficiency was estimated. Consequences of Bt-CBP21 inclusion in the chitinase reaction was different depending upon the source of chitinase. Bt-CBP21 enhanced V_max_ of bacillar chitinase by seven folds without any change in K_m_. In contrast, the effect on V_max_ or K_m_ of *H. armigera* chitinase activity was negligible (Table 1). The synergistic effect of CBP on chitinase has been reported from other microbial systems also [5], [7]. The conformational changes upon CBP interaction with chitinase, impart catalytic activity as demonstrated in *B. licheniformis* [24]. To understand such diverse consequences of Bt-CBP21 on bacillar or *Helicoverpa* chitinase, we examined the structural features of both the chitinases. Except for the size and position of characteristic domains, the bacillar and *Helicoverpa* chitinase are largely identical. Distinctively, chitinase of *H. armigera* has an additional carbohydrate-binding module (CBM) (Figure 3). The exact functional consequence of existence of CBM is not clear at present. Nevertheless, the observed positive kinetic interaction between Bt-CBP21 and bacillar chitinase may facilitate enhanced chitin degradation. It has been suggested that synergism is a consequence of CBPs binding with insoluble crystalline substrate leading to structural alterations and higher access to chitinases [25]. This is in agreement with reported enhancement of chitinase activity upon interaction with CBPs [24]. The degraded chitin may serve as growth nutrient and further help in growth and proliferation of Bt. In addition to examine the interaction between Bt-CBP21 and chitinase, we also examined its interaction with insecticidal protein, Cry1Ac. The structure and function of Domain III of Cry toxins has been compared to a number of different proteins but its similarity to CBMs found in glycoside hydrolases, lyases and esterases is particularly striking [26], [27]. Such similarity prompted speculation that the binding of Cry1Ac to carbohydrate moiety is crucial in exerting its biological activity [26]. Further, it has been shown that there is significant structural homology between CBM and Domain III of Cry1Aa (ca. 95% homologous to Cry1Ac) which probably facilitates binding of toxin [26]. To evaluate such phenomenon, the interaction of Bt-CBP21 with Cry1Ac was investigated by (i) monitoring gross structural changes by circular dichroism and (ii) *in vitro* pull down assays. Bt-CBP21 and Cry1Ac were purified to homogeneity and their CD spectra were recorded. As reported earlier, the CD spectrum of Cry1Ac revealed alpha-helices and beta-sheets at a ratio of 1∶2 with significant randomness in its secondary structure (Figure 4) [28]. The CD spectrum of Bt-CBP21 exhibited predominance of beta strands and significant amount of randomness (Figure 4). The combined spectrum of Bt-CBP21 and Cry1Ac revealed similar structure with overall reduction in randomness of both proteins, clearly revealing an interaction between the two proteins (Figure 4). The interaction between Cry1Ac and Bt-CBP21 was further examined by *in vitro* pull down assays (Figure 5). Since Bt-CBP21 was expressed with His-tag, it was purified by affinity chromatography. The purified Bt-CBP21 was allowed to bind to Ni-NTA resin. After several washes with buffer, purified Cry1Ac was passed through the matrix. Elution with histidine counter-ion and subsequent western blot analysis of eluate revealed co-eluting Cry1Ac-Bt-CBP21 conjugate suggesting that the two proteins interact. Taken together, results of CD spectroscopy and *in vitro* pull down assay experiments also revealed interaction between these polypeptides. Next, to understand the functional significance of interaction of Cry1Ac with Bt-CBP21 in mediating insecticidal activity, we examined direct binding of Cry1Ac with chitin at various physiological pH encountered through larval gut namely, pH 5.5, 7.5 and 10.5. To examine such interaction as function of pH, purified protoxin, Cry1Ac (130 kDa) was incubated with colloidal chitin and after washing with buffer of desired pH, the bound Cry1Ac was eluted with guanidine hydrochloride and its amount estimated. It is evident from results in Figure 6 that maximum amount of Cry1Ac remained bound to chitin at pH 10.5. This pH (10.5) corresponds to the reported midgut pH of *Helicoverpa* larvae. In the context of binding of Cry1Ac to the receptor at the gut epithelium the common binding of Bt-CBP21/Cry1Ac to chitin at pH 10.5 assumes functional significance. The enriched concentration of Cry1Ac/Bt-CBP21 probably facilitates toxin action. The chitin interaction of Bt-CBP21 may be a consequence of presence of several aromatic residues specifically, tyrosine and tryptophan [22]. Interestingly, the aromatic amino acid residues important for binding to carbohydrate structure are not conserved in Cry proteins [26]. Thus, it is tempting to speculate that the aromatic residues of Bt-CBP21 facilitate enrichment of Cry1Ac protein in the vicinity of the receptor at the epithelial tissue of larvae.

**Table 1:** Kinetic parameters of Bt-Chitinase with Bt-CBP21.

Interacting proteins	K_m_ (µM)	V_max_ (nmoles/min/mg)
Bt-Chitinase	30	45
Bt-Chitinase +Bt-CBP21	27.5	180
Ha-Chitinase	1.4	4.55
Ha-Chitinase+Bt-CBP21	1.6	5.9

The enzyme activity was measured with 50 µM of the substrate, chitobioside, 4-MU-(GlcNAc)2 in 50 mM sodium-phosphate buffer, pH 7.0 containing purified Bt-Chitinase or Ha-Chitinase and purified Bt-CBP21. Reaction was carried out at 30°C for 20 min. K_m_ and V_max_ were calculated. Bt-CBP21 did not display any chitinolytic activity.

**Figure 7:**
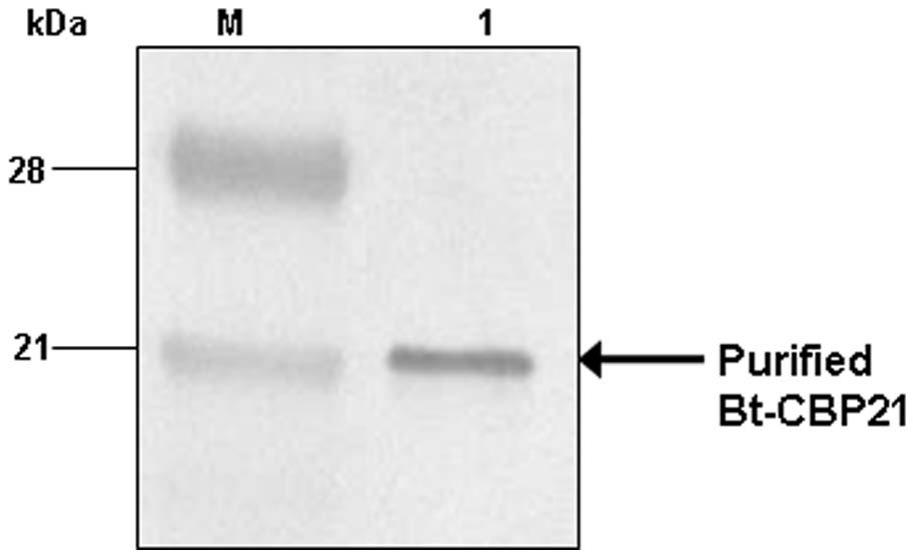
Localization of Bt-CBP21 in Bt cultures and immunodetection with anti-Bt-CBP21 antibodies. Spore-crystal suspension of Bt-HD1 was resolved on Renograffin gradient and crystals were solubilized with 50 mM sodium-carbonate buffer. The solubilized crystal proteins were resolved on 12% SDS-PAGE and immunoblotted. Lane 1: Protein standard marker (in kilodaltons). Lane 2: Crystal proteins.

Our proposed role of Bt-CBP21 as facilitation of Cry1Ac adherence at the epithelial layer of midgut is strengthened further by investigation into its site of accumulation in bacteria. Fractionation of sporulating cultures of Bt*-*HD1 by renograffin density gradient resulted in recovery of insecticidal protein containing crystals free of cellular debris. Resolution of crystal proteins on SDS-PAGE and screening with antibodies raised against Bt-CBP21 revealed its occurrence in the crystal protein complex (Figure 7). These observations established that Bt-CBP21 co-localises with Cry1Ac in the spore mother cell. It is likely that the ingestion of Bt-CBP21 along with Cry1Ac by the insect larvae may facilitate localized enrichment of the protoxin at the gut epithelial receptors.

The co-localization of Bt-CBP21 with Cry1Ac in spore mother cell and their interaction prompted us to examine effect of Bt-CBP21 on the insecticidal activity of Cry1Ac against larvae of *H. armigera*. To examine this possibility further, Bt-CBP21 was included in diet along with purified Cry1Ac and fed to neonate larvae. After 48 h, the mortality of larvae was scored. Bt-CBP21 alone did not affect the growth and development of larvae. However, in the presence of Cry1Ac, Bt-CBP21 potentiated Cry1Ac effect (T-Test; P-value<0.05) (Table 2, Figure S2).

**Table 2:** Assessment of insecticidal activity of Cry1Ac in the presence of Bt-CBP21 against neonate larvae of *Helicoverpa armigera*.

Treatment	LC_50_ values (µg/ml)	Confidence Interval (95%)
		(Lower–Upper limit)
Cry1Ac alone	46.9	43.6–49.9
Cry1Ac+Bt-CBP21	38.8	35.8–41.5

Initial number of insects (n) for each bioassay is 60.

In addition to the speculated role in facilitating action of Cry1Ac protein, we examined the impact of Bt-CBP21 in the ecological niche of Bt. The natural niche of Bt is either the phylloplane of plants, insect cadavers or soil. In all the three likely environments, the bacterium faces competition with incidental microflora. To investigate the successful propagation of Bt spores, the effect of Bt-CBP21 was examined on the growth of certain fungi. Bt-CBP21 exhibited potent antifungal effect on *C. oryzae, A. oryzae, A. parasiticus, A. flavus and V. dahliae* (Table 3). Further, inclusion of Bt-Chitinase with Bt-CBP21 enhanced the fungistatic effect. CBPs displaying antifungal activity have been isolated from plants, bacteria and fungi [29]. These proteins inhibit fungi by mechanism similar to pathogenesis related proteins by binding to cell wall chitin and disrupting cell polarity, thus leading to inhibition of fungal growth [29], [30].

**Table 3:** Antifungal activity of Bt-CBP21.

Fungal Strains	Amount ofBt-CBP21	Percentageinhibition
	(in µg)	(after 2 days)
*Curvularia oryzae*	10	50%
	20	80%
*Aspergillus oryzae*	2.5	70%
	5.0	90%
*Aspergillus parasiticus*	2.5	70%
	5.0	90%
*Verticillium dahliae*	2.5	80%
	5.0	80%

Antifungal assays were performed using purified Bt-CBP21. Approximately, 100 spores from each fungal strain were seeded on Potato-dextrose medium. Growth inhibition was recorded in response to 2.5, 5, 10 and 20 µg of Bt-CBP21 for 2 days.

Taken together, our results on the characterization of Bt-CBP21 suggest that it may facilitate adherence of Cry1Ac at the midgut epithelial tissue of larvae and in the ecological niche it may offer the bacterium, rapid proliferation advantage by virtue of its fungistatic action.

### Conclusions

Bt*-*HD1 synthesizes Bt-CBP21 that accumulates in the spore mother cell. The Bt-CBP21 interacts with Cry1Ac and potentiates insecticidal activity. In addition, it is fungistatic against certain fungi. We speculate that Bt-CBP21 facilitates insecticidal action of Cry1Ac by binding to chitin at midgut epithelial cells.

## Supporting Information

Figure S1 **Multiple alignment of Bt-CBP21.** Bt-CBP21 was aligned with other proteases using ClustalW (www.ebi.ac.uk) on the basis of hydrophobicity [EMBL accession no. Q816G5 and Q81GC6, Camelysin of *Bacillus cereus*; Q8GJ76, Camelysin precursor *of B. cereus*; Q81TI3, Putative spore coat associated protein of *Bacillus anthracis*; Q8ESH2, Putative spore coat associated protein of *Oceanobacillus iheyensis*; Q8KNQ1, Putative spore coat associated protein of *B. thuringiensis (subsp. israelensis)*; Q8ERK0 and Q8ES42, Spore coat associated protein of *O. iheyensis*; Q81GC8, Spore coat associated protein of *B. cereus*; Q81TI4, Spore coat associated protein of *B. anthracis*; Q8ENF4, Spore coat associated protein of *O. iheyensis*; Q9KB07, Spore coat associated protein of *Bacillus halodurans*]. Amino acids which are conserved are shaded. Dashes indicate gaps left to improve the alignment. All the sequences are starting from Met-1 of the peptide. The tryptophan residues (W-104, W-106, W-138, W-153, W-167), Histidine (H-97), Tyrosine (Y-80,Y-114, Y-151), Proline (P-52, P-63, P-112, P-116, P-136), Phenylalanine (F-20, F-21, F-31, F-61, F-94, F-102, F-134, F-156, F-158, F-168, F- 179) which are highly conserved are shaded.
(TIF)

Figure S2 **Bioassay for assessment of Cry1Ac insecticidal activity in the presence of Bt-CBP21.** *H. armigera* neonates were reared on artificial diet as described in Materials and methods. Varying concentrations of purified Cry1Ac and 20 µg/ml of purified Bt-CBP21 were mixed with artificial diet and fed to the neonates. Twenty neonates were used for each treatment and each assay was performed thrice. Mortality was recorded 48 h post-treatment.
(TIF)
