# Supplementary figures and images for: Characterization of a Chitin-Binding Protein from Bacillus thuringiensis HD-1
Source: PLoS One. 2013 Jun 18;8(6):e66603. doi: 10.1371/journal.pone.0066603 (PMC3688941; doi:10.1371/journal.pone.0066603)

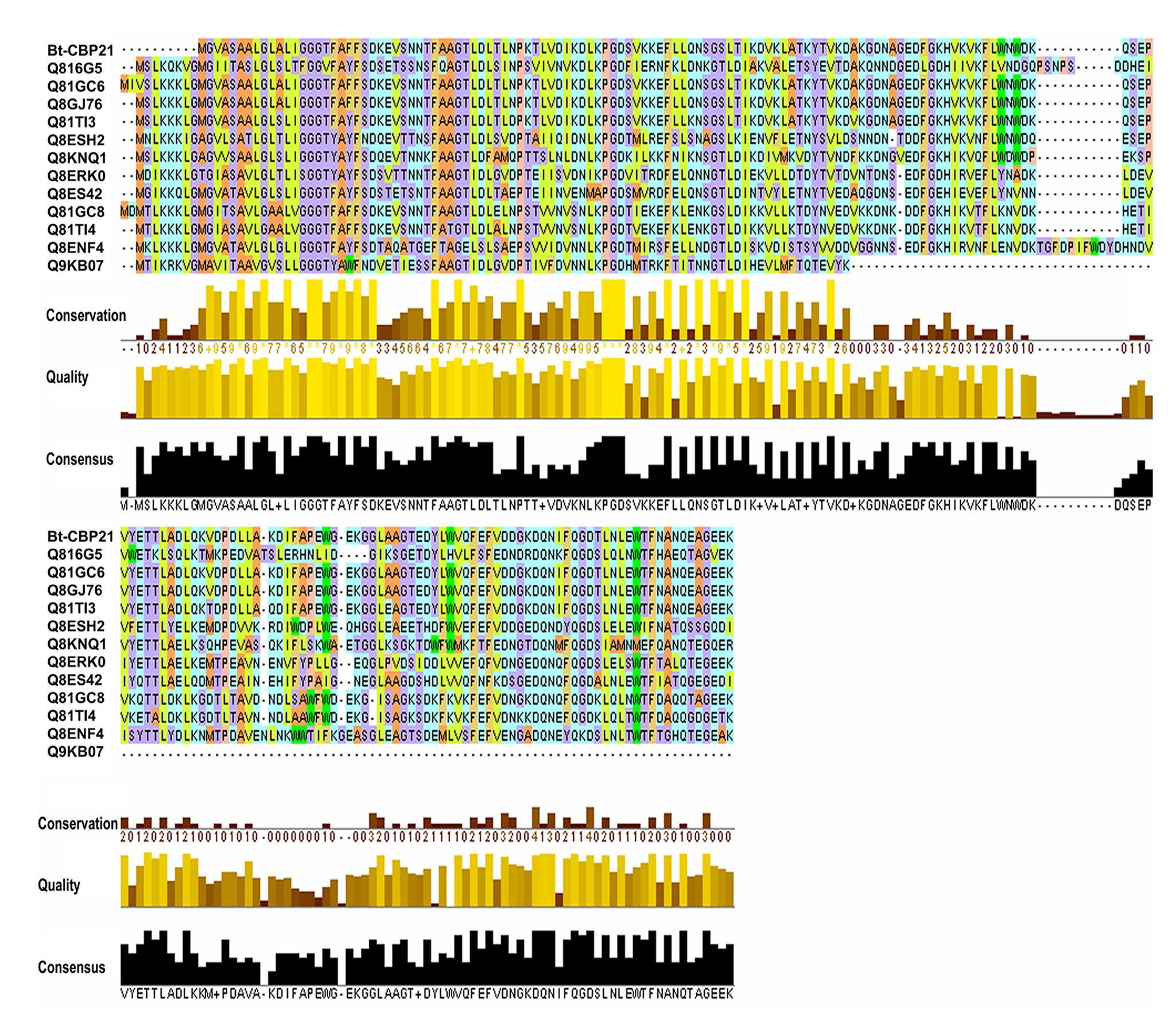

Supplement: Figure S1 — Multiple alignment of Bt-CBP21. Bt-CBP21 was aligned with other proteases using ClustalW (www.ebi.ac.uk) on the basis of hydrophobicity [EMBL accession no. Q816G5 and Q81GC6, Camelysin of Bacillus cereus; Q8GJ76, Camelysin precursor of B. cereus; Q81TI3, Putative spore coat associated protein of Bacillus anthracis; Q8ESH2, Putative spore coat associated protein of Oceanobacillus iheyensis; Q8KNQ1, Putative spore coat associated protein of B. thuringiensis (subsp. israelensis); Q8ERK0 and Q8ES42, Spore coat associated protein of O. iheyensis; Q81GC8, Spore coat associated protein of B. cereus; Q81TI4, Spore coat associated protein of B. anthracis; Q8ENF4, Spore coat associated protein of O. iheyensis; Q9KB07, Spore coat associated protein of Bacillus halodurans]. Amino acids which are conserved are shaded. Dashes indicate gaps left to improve the alignment. All the sequences are starting from Met-1 of the peptide. The tryptophan residues (W-104, W-106, W-138, W-153, W-167), Histidine (H-97), Tyrosine (Y-80,Y-114, Y-151), Proline (P-52, P-63, P-112, P-116, P-136), Phenylalanine (F-20, F-21, F-31, F-61, F-94, F-102, F-134, F-156, F-158, F-168, F- 179) which are highly conserved are shaded. (TIF) [file pone.0066603.s001.tif]

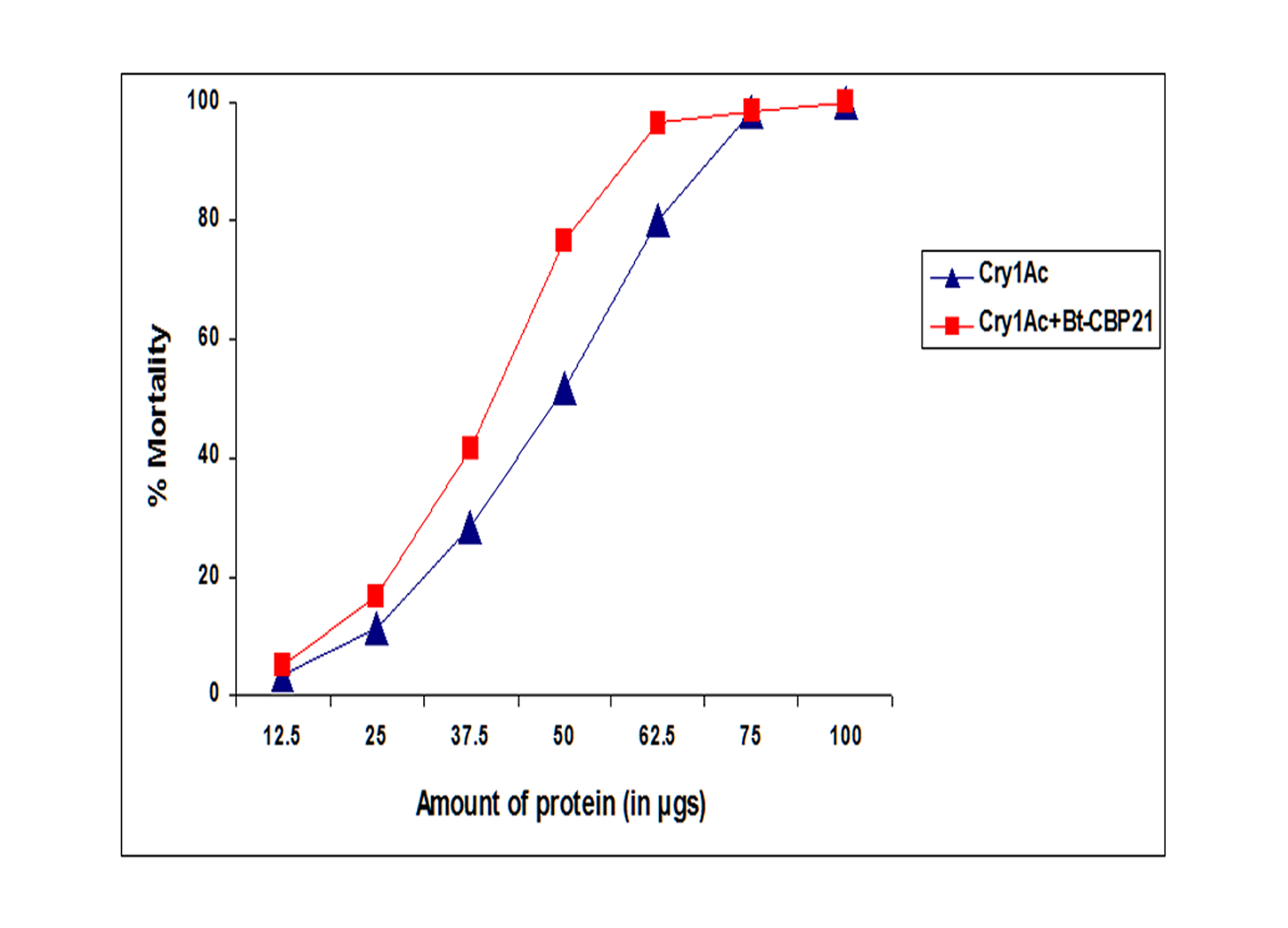

Supplement: Figure S2 — Bioassay for assessment of Cry1Ac insecticidal activity in the presence of Bt-CBP21. H. armigera neonates were reared on artificial diet as described in Materials and methods. Varying concentrations of purified Cry1Ac and 20 µg/ml of purified Bt-CBP21 were mixed with artificial diet and fed to the neonates. Twenty neonates were used for each treatment and each assay was performed thrice. Mortality was recorded 48 h post-treatment. (TIF) [file pone.0066603.s002.tif]
